# Depletion of B220^+^NK1.1^+^ cells enhances the rejection of established melanoma by tumor-specific CD4^+^ T cells

**DOI:** 10.1080/2162402X.2015.1019196

**Published:** 2015-04-01

**Authors:** Kyle A Wilson, Stephen R Goding, Harold R Neely, Kristina M Harris, Paul Andrew Antony

**Affiliations:** 1 Program in Molecular Microbiology and Immunology; University of Maryland School of Medicine; Baltimore, MD USA; 2 Department of Pathology; University of Maryland School of Medicine; Baltimore, MD USA; 3 Department of Microbiology and Immunology; University of Maryland School of Medicine; Baltimore, MD USA; 4 Immune Tolerance Network; Bethesda, MD USA; 5 Tumor Immunology and Immunotherapy Program; University of Maryland Cancer Center; Baltimore, MD USA

**Keywords:** adoptive cell transfer, CD4^+^ T cells, IKDC, NK cells, melanoma, Pre-mNK cells

## Abstract

Five-year survival rates for patients diagnosed with metastatic melanoma are less than 5%. Adoptive cell transfer (ACT) has achieved an objective response of 50% by Response Evaluation Criteria in Solid Tumors (RECIST) in this patient population. For ACT to be maximally effective, the host must first be lymphodepleted. It is hypothesized that lymphodepletion may remove regulatory elements and cytokine sinks, or increase the activation and availability of antigen presenting cells (APCs). We use an *in vivo* model to study the ACT of tumor-associated antigen (TAA)-specific CD4^+^ T cells (TRP-1 cells). We have discovered that depletion of NK1.1^+^ cells enhances the rejection of established melanoma tumors by adoptively transferred TRP-1 CD4^+^ T cells. NK1.1^+^ cell depletion increases the number of CD4^+^ T cells, the serum concentration of pro-inflammatory cytokines, autoimmune vitiligo, host survival and prevented recurrence after ACT. Because multiple cells express NK1.1, we targeted different NK1.1^+^ cell populations using antibodies specific for NK cells, pre-mNK cells, and innate lymphoid cells (ILCs). Our data suggests that NK1.1^+^B220^+^ pre-mNK cells (also known as interferon-producing killer dendritic cells; IKDCs) are an important inhibitor of the CD4^+^ T cell response to melanoma. Understanding this mechanism may help design new immunotherapies to modulate the activity of pre-mNKs in the face of an antitumor immune response and inhibit their suppression of adoptively transferred T cells.

## Introduction

The incidence of melanoma has been increasing at a faster rate than any other malignancy over the past three decades.^1^ The lifetime probability of developing melanoma of the skin in the United States is 1 in 34 for males and 1 in 53 for females Presently, there are five FDA-approved therapies for metastatic melanoma: dacarbazine (DITC), high-dose IL-2, ipilimumab, pembrolizumab, and nivolumab. The objective response rates (ORRs) for these therapies are 15%–25%^2^, 16%^3,4^, 11%^5^, 26%^6^, and 25%^7^ respectively.^8^ The ORRs for these therapies appear to increase if they are used as part of a combination therapy. Randomized trials to formally test this hypothesis are underway.

ACT is an experimental therapy that has had much success in treating melanoma in mice and humans. During ACT, either cytotoxic CD4^+^ or CD8^+^ T cells that recognize a melanoma antigen are infused into the host.^9-18^ It was observed that preconditioning of the host with a non-myeloablative regimen prior to ACT improved the outcome of therapy.^17^ Lymphodepletion enhances ACT in multiple ways, including (1) the elimination of regulatory T cells (Tregs)^12^, (2) the elimination of cells that use the same resources as the adoptively transferred T cells, termed *cytokine sinks*,^19^ and (3) enhancing the availability and activation of APCs, presumably by allowing more pathogen associated molecular patterns (PAMPs) to cross the compromised gut endothelial barrier.^20,21^ A study in mice found a positive correlation between the dose of radiation prior to ACT and therapeutic efficacy.^22^ Another study in humans found that patients receiving only non-myeloablative therapy had an ORR of 49%, while patients receiving an additional 2 gray (Gy) or 12 Gy of total body irradiation (TBI) had ORRs of 52% and 72%, respectively. This implies a correlation between the intensity of the lymphodepletion regimen and the effectiveness of ACT,^23^ suggesting that the increased intensity removes another cell population involved in regulating the immune response.

Previously, we showed that ACT of naive CD4^+^ T cells into lymphopenic hosts could treat large, established tumors.^11^ The naive CD4^+^ T cells differentiated *in vivo* to Th1 cytotoxic T cells and rejected established melanoma in both irradiated wild-type (WT)^10^ and RAG^−/−^ mice.^11^ To determine the mechanism of tumor rejection, we sought to deplete specific cell subsets by using antibodies and genetic knockouts. We were particularly interested in NK cells because these cells have been shown to synergize with CD4^+^ T cells to reject tumors.^24^ However, our previous data suggested that depletion of NK cells with antibodies to NK1.1 had no affect on tumor immunity^11^ except for anecdotal evidence that mice acquired autoimmune vitiligo faster than animals that only received TRP-1 cells. In contrast to these observations, ACT of TRP-1-specific CD4^+^ T cells did not efficiently reject tumors in RAG^−/−^γ_c_^−/−^ hosts, which lack NK cells.^11^ This was due to interrupted γ_c_-signaling on the host DCs, resulting in decreased IL-12 production by the DCs^25^ and inefficient Th1 differentiation of the infused, naive CD4^+^ T cells, as shown by a loss of Tbet expression.^11^ Adoptive transfer of NK cells into RAG^−/−^γc^−/−^ mice caused no further reduction in efficacy, demonstrating that there was some other defect related to γ_c_-signaling and rendering the results equivacol.^11^

The discovery of another NK1.1^+^ cell in C57BL/6 mice further confounded these observations.^26,27^ These cells, initially named IKDCs, were later reclassified as pre-mNK cells, and are thought to be a lineage of NK cells.^28,29^ Pre-mNK cells express many of the same markers as *bona fide* NK cells, including NK1.1, NKp46, NKG2D, FASL, and CD49b (DX5). They also express markers more commonly associated with APCs, including B220, MHC class II, CD11c, and B7–1.

Because γ_c_-signaling on DCs in RAG^−/−^γ_c_^−/−^ hosts is defective, it was impossible to determine the effect of NK cells or pre-mNK cells on TRP-1-specific CD4^+^ T cells in this model. While RAG^−/−^γ_c_^−/−^ hosts lack pre-mNK and NK cells, owing to their dependence on IL-15,^28^ both are present in RAG^−/−^ hosts. Thus, we sought to dissect the contribution of these cellular populations on antitumor immunity by using antibodies and genetic knockouts to specifically target each cell population.

NK1.1^+^ cells have been shown to contribute to antitumor immunity in some models.^24,26,27^ In others, they have been observed to prevent autoimmunity, GVHD, and tumor immunity, and cause chronic exhaustion of T cells.^30-36^ Since tyrosinase related protein-1 (TRP-1) is a TAA and depletion of NK1.1^+^ cells leads to enhanced autoimmunity and increased T cell activity, we investigated the possibility that pre-mNK cells could be preventing antitumor immunity in our model.

## Results

### Depletion of NK1.1^+^ cells enhances tumor rejection and prevents recurrence

**Figure 1.**
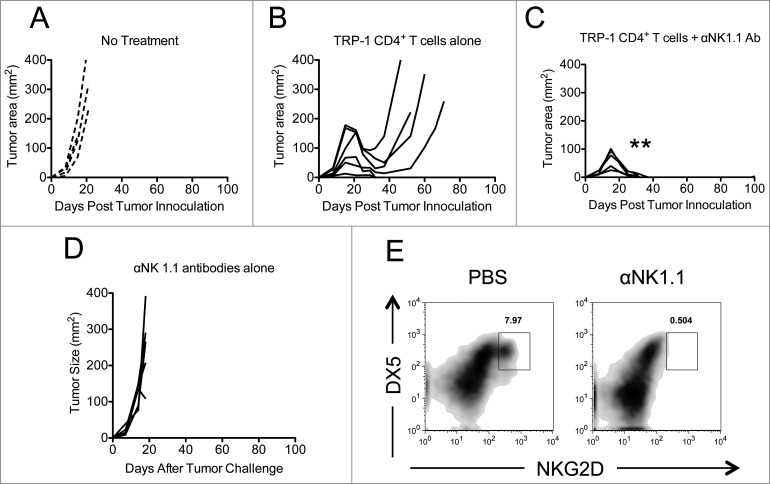
NK1.1^+^ cell depletion enhances the rejection of established melanoma by adoptively transferred TRP-1-secific CD4^+^ T cells. RAG^−/−^ mice were inoculated subcutaneously with 3×10^5^ B16.F10 on day 0 and left untreated (**A**), received ACT of 5×10^4^ TRP-1-specific CD4^+^ T cells on day 7 (**B**), or received ACT on day 7 plus 200 μg of anti-NK1.1 intraperitoneally on days 0, 7, and 14 (**C**). A, B, and C show tumor area as a function of time post tumor inoculation for each mouse in each experimental group. **, *p* < 0.0001. (**D**) Anti-NK1.1 antibody alone has no effect on the rejection of established melanoma. RAG^−/−^ mice were inoculated subcutaneously with 3×10^5^ B16.F10 on day 0 and left untreated or received 200 μg of anti-NK1.1 intraperitoneally on days 0, 7, and 14. (**D**) Tumor area as a function of time for each replicate of each experimental group is plotted. (**E**) Anti-NK1.1 antibody depletes NK cells. Splenocytes from each indicated experimental group were harvested at day 21 post tumor inoculation and analyzed for the presence of NK cells and pre-mNK cells.

Previously we and others have published that NK cell depletion by anti-NK1.1 antibodies had no affect on tumor immunity^10,11^ after ACT of TRP-1 CD4^+^ T cells into lymphopenic hosts. However, anecdotally, it appeared to increase autoimmune vitiligo (*data not shown*). We modified our ACT model such that tumor rejection was suboptimal to determine if NK1.1^+^ cell depletion would enhance tumor rejection and survival, as it had enhanced autoimmunity. NK1.1^+^ cells were depleted by administering anti-NK1.1 antibody (PK136) on days 0, 7, and 14 after tumor inoculation. Adoptive transfer of 5 × 10^4^ naive TRP-1-specific CD4^+^ T cells into lymphopenic RAG^−/−^ tumor-bearing hosts was performed by tail vein injection on day 7, as previously described.^9,11^ Individual mice were followed and each replicate is shown with controls (**Fig. 1**). Administration of anti-NK1.1 antibody enhanced tumor rejection, as shown by complete resolution of tumors (**Fig. 1C**) when compared to controls (**Fig. 1A, B**). However, mice receiving anti-NK1.1 antibodies alone had no tumor resolution, despite the fact that NK cells were depleted, as shown by NKG2D and DX5 staining (**Fig. 1D and E**).

**Figure 2.**
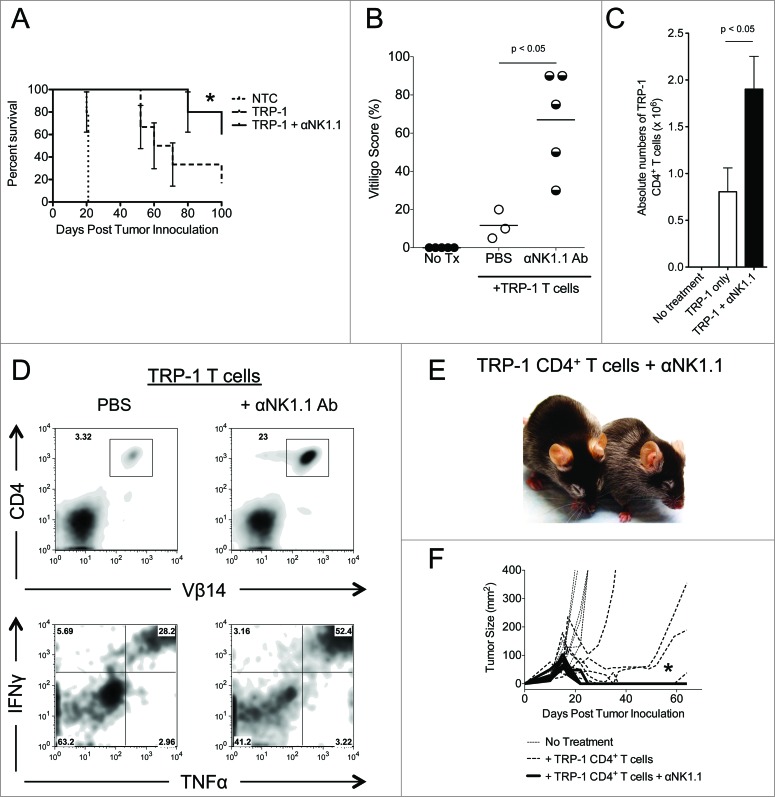
NK1.1^+^ cell depletion enhances survival and autoimmune vitiligo, increases the effector function of TRP-1 CD4^+^ T cells, and prevents recurrence of melanoma. (**A**) Percent survival for each of the aforementioned experimental groups was plotted as a function of time post tumor inoculation. *, *p* < 0.05. (**B**) Percent of body area affected by vitiligo was determined for each experimental group 35 d post tumor inoculation, *, *p* < 0.05. (**C**) 17–21 d post tumor inoculation, spleens were harvested from animals in each experimental group, made into a single cell suspension, and stained with anti-CD4. The absolute number of CD4^+^ T cells isolated from each spleen was determined, *p* < 0.05. (**D**) Splenocytes were stained for the indicated markers. (**E**) Mice receiving anti-NK1.1 in addition to ACT experienced patchy, irregular, vitiligo earlier than animals receiving ACT alone. (**F**) Tumor areas from each experimental group plotted as a function of time show that animals receiving anti-NK1.1 in addition to ACT experience tumor relapse at a significantly lower rate than animals receiving ACT alone, *p* < 0.05.

In addition to complete resolution of tumors, there was also improved overall survival (**Fig. 2A**). Since TRP-1 is expressed in normal melanocytes as well as B16.F10 melanoma, all animals receiving ACT of TRP-1-specific CD4^+^ T cells eventually experienced some autoimmune vitiligo, characterized by a patchy, irregular loss of pigmentation (**Fig. 2B and E**).^11^ However, mice receiving anti-NK1.1 therapy in addition to ACT of naive TRP-1 CD4^+^ T cells experienced more vitiligo by day 35 after treatment (**Fig. 2B**). Congruent with these results, there were increased absolute numbers and frequency of TRP-1-specific CD4^+^ T cells present in mice receiving anti-NK1.1 antibodies plus TRP-1 CD4^+^ T cells (**Fig. 2C and D**). The TRP-1-specific CD4^+^ T cells also expressed higher amounts of IFNγ and TNFα when compared to control groups (**Fig. 2D**).

Previously, we had observed that melanoma recurred about 50% of the time in this model.^9,37^ In these cases, the CD4^+^ T cells had become exhausted, expressing high amounts of PD-1, LAG-3, and TIGIT.^9^ Tumor-specific Tregs also increased in number and proportion during recurrence.^9^ Since NK1.1^+^ cell depletion has been shown to decrease chronic exhaustion of T cells,^35^ we wondered if NK1.1^+^ cell depletion would prevent exhaustion of CD4^+^ T cells and thus prevent recurrence. Mice were followed for at least 100 d post tumor inoculation. Mice treated with anti-NK1.1 antibodies had fewer tumor relapses relative to animals receiving ACT alone (**Fig. 2F** **and** **Table 1**). The majority of animals receiving anti-NK1.1 antibody therapy in addition to ACT were essentially cured for the duration of this study with few relapses occurring past 100 d post tumor inoculation. Together, these data demonstrate that the depletion of NK1.1^+^ cells enhances the rejection of established melanoma by adoptively transferred TRP-1-specific CD4^+^ T cells. In addition, it increases survival and autoimmune vitiligo, and prevents recurrence of melanoma.

**Table 1.** Recurrence rates compared between mouse strains and different antibody therapies. Recurrence rates represent the percentage of mice that had recurred during the first 100 d after therapy

Mouse strain	Therapy	Recurrence rate
RAG^−/−^	TRP-1 only	52%
RAG^−/−^	TRP-1 + anti-NKl.l	21%
RAG^−/−^	TRP-1 + anti-AsialoGMl	77%
RAG^−/−^	TRP-1 + anti-B220	28%
RAG^−/−^	TRP-1 + anti-Thyl.2	75%
RAG^−/−^IL-15^−/−^	TRP-1 only	25%

### Depletion of NK1.1^+^ cells increases the serum concentration of pro-inflammatory cytokines

**Figure 3.**
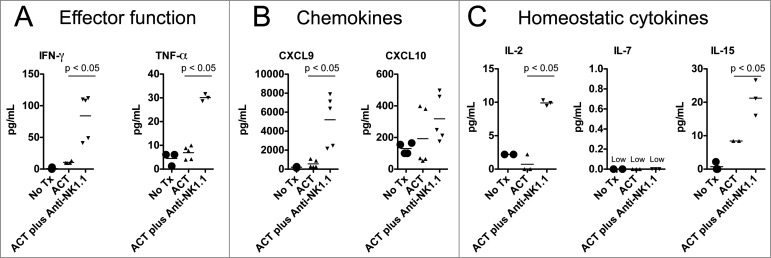
NK1.1^+^ cell depletion in addition to ACT increases the serum concentration of pro-inflammatory cytokines. On day 14 post tumor inoculation, serum was harvested from each experimental group, and analyzed by Milliplex for the concentration of (**A**) IFNγ and TNFα, (**B**) CXCL9 and CXCL10 and (**C**), IL-2, IL-7, and IL-15.

To further investigate the effects of NK1.1^+^ cell depletion on the adoptively transferred TRP-1-specific CD4^+^ T cells, we measured the serum concentration of certain pro-inflammatory cytokines. As we have noted, mice receiving anti-NK1.1 antibodies have an increased frequency of IFNγ^+^ TNFα^+^ TRP-1-specific T cells (**Fig. 2D**). Consistent with this observation, NK1.1^+^ cell depletion also leads to higher concentrations of TNFα and IFNγ, pro-inflammatory chemokines, CXCL9 and CXCL10, and homeostatic cytokines, IL-2 and IL-15, in the serum of host animals (**Fig. 3**). The increased concentrations of CXCL9 and CXCL10 were consistent with previous results.^11^ The elevated IL-2 and IL-15 concentrations may indicate that anti-NK1.1 antibody depleted cytokine sinks, namely NK cells, which express the IL-2/15Rβγ chains. IL-7 was universally low, which may reflect the consumption of this cytokine by CD4^+^ T cells.^38^ These results show that NK1.1^+^ cell depletion leads to an increase in the serum concentration of pro-inflammatory and homeostatic cytokines that may reflect enhanced activation of TRP-1-specific CD4^+^ T cells.

### Depletion of NK cells does not enhance tumor rejection to the same extent as NK1.1^+^ cell depletion

**Figure 4.**
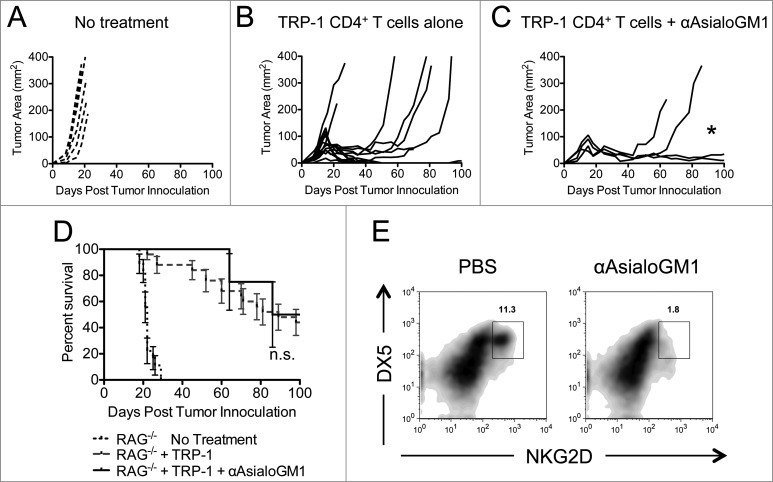
Asialo-GM1^+^ cell depletion does not enhance the rejection of established melanoma to the same extent as NK1.1^+^ cell depletion. RAG^−/−^ mice were inoculated subcutaneously with 3×10^5^ B16.F10 on day 0 and left untreated (**A**), received ACT of 5×10^4^ TRP-1-specific CD4^+^ T cells on day 7 (**B**), or received ACT on day 7 plus 20 μL of anti-Asialo-GM1 intraperitoneally on days 2, 0, 7, and 14 (**C**). A, B, and C show tumor area as a function of time post tumor inoculation for each mouse in each experimental group. *, *p* < 0.05. (**D**) Percent survival for each of the aforementioned experimental groups was plotted as a function of time post tumor inoculation. There is no significant difference between the survival of mice treated with TRP-1 cells vs. mice treated with TRP-1 cells plus anti-Asialo-GM1, *p* > 0.05. (**E**) On day 21 post tumor inoculation, splenocytes were isolated from the indicated experimental groups and analyzed for the presence of NK cells.

It has been shown that depletion of NK cells can enhance tumor-specific CD8^+^ T cell rejection of B16 tumors^19^ and prevent chronic exhaustion of CD8^+^ T cells in a viral model.^35^ Furthermore, depletion of NK cells is known to enhance pathogenic CD4^+^ T cell activity in a graft versus host disease model. It could be that NK depletion removes a cytokine sink, and liberates IL-2, IL-7, or IL-15 – all of which could alleviate T cell exhaustion and help expand CD4^+^ T cells *in vivo*. Alternatively, NK cells could be killing activated CD4^+^ T cells using a death receptor such as FasL, TRAIL, or an NKG2- type ligand.^36,39,40^ Because NK1.1 is expressed on multiple cell types, it was necessary to deplete NK cells by another method to rule out their potential role in our model. We chose to target NK cells with an antibody that recognizes Asialo-GM1. Asialo-GM1 is a ganglioside that is known to be expressed on NK cells.^41^ Anti-Asialo-GM1 was injected similarly to anti-NK1.1 antibody, as in **Fig. 1**. Mice receiving anti-Asialo-GM1 antibody in addition to ACT still experienced tumor recurrence, although to a lesser extent than animals receiving TRP-1-specific CD4^+^ T cells alone (**Figs. 4A, B and C**). Survival was not enhanced over ACT alone (**Fig. 4D**). To confirm that NK cells were depleted, we stained for NKG2D^+^DX5^+^ cells (**Fig. 4E**). Lastly, some authors have observed that ILCs express NK1.1 and Thy1.2. ILCs were depleted with anti-Thy1.2 antibodies but there was no effect on tumor immunity (**Fig. S1**). These data show that, while NK cells may be behaving as cytokine sinks in our model, the antitumor activity of anti-NK1.1 antibody cannot solely be attributed to the removal of NK cells or ILCs.

### Fc receptor activation does not explain enhanced tumor immunity

**Figure 5.**
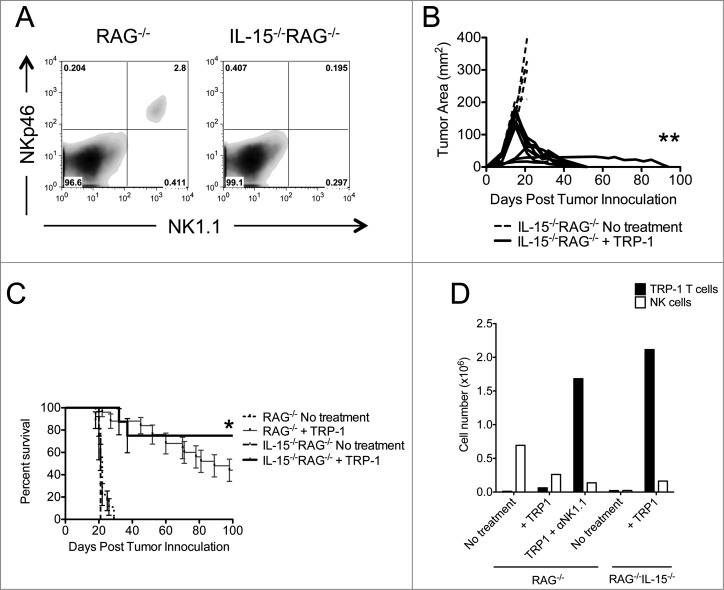
Tumor therapy is enhanced in IL-15^−/−^RAG^−/−^ mice. (**A**) Splenocytes from each indicated experimental group were harvested at day 21 post tumor inoculation and analyzed for the presence of NK cells and pre-mNK cells. (**B**) IL-15^−/−^RAG^−/−^ mice were inoculated subcutaneously with 3×10^5^ B16.F10 on day 0 and left untreated or received ACT of 5×10^4^ TRP-1-specific CD4^+^ T cells. Tumor area as a function of time for each replicate of each experimental group is plotted. Compared to RAG^−/−^ historical controls, **, *p* < 0.0001. (**C**) Percent survival for each of the aforementioned experimental groups was plotted as a function of time post tumor inoculation. Survival differences between RAG^−/−^ and IL-15^−/−^RAG^−/−^ treated with TRP-1 cells are significantly different, *, *P* < 0.05. (**D**) Absolute cell numbers of TRP-1 CD4^+^ T cells and NK cells from individual groups (RAG^−/−^ vs. IL-15^−/−^RAG^−/−^) as shown 7 d after adoptive T cell transfer.

Because the Fc region of the depleting antibodies used in our previous experiments could signal through Fc receptors,^42^ thus enhancing antitumor inflammation, we repeated ACT of TRP-1-specific CD4^+^ T cells in IL-15^−/−^RAG^−/−^ hosts. IL-15 controls the homeostasis of CD8^+^ memory T cells and NK cells. We acquired IL-15^−/−^RAG^−/−^ mice because these animals lack these cellular subsets in addition to CD4^+^ T cells and B cells. We confirmed that NK cells were absent in IL-15^−/−^RAG^−/−^ mice (**Fig. 5A**). Therefore, NK1.1^+^ cell depletion by means of anti-NK1.1 antibody would be superfluous, allowing us to eliminate the confounding effects of Fc receptor signaling in this system. IL-15 has been shown to have multiple and contradictory effects on the homeostasis of naive CD4^+^ T cells,^43,44^ and recent data show that anti-viral memory CD4^+^ T cells may use IL-15 *in vivo*.^45^ Therefore, we wanted to confirm in our model whether IL-15 had any effects on the homeostasis of naive CD4^+^ T cells and subsequent long-term maintenance of treatment. Assuming that ACT of TRP-1-specific CD4^+^ T cells could reject established melanoma tumors in IL-15^−/−^RAG^−/−^ mice, we could use this model to dissect the activity of NK cells on adoptively transferred CD4^+^ T cells without also activating Fc receptors. We found that ACT of 5×10^4^ naive TRP-1-specific CD4^+^ T cells into IL-15^−/−^RAG^−/−^ hosts leads to complete rejection of established B16.F10 tumors and increased survival relative to RAG^−/−^ hosts (**Figs. 5B and 5C**). Consistent with NK1.1 cell depletion in RAG^−/−^ hosts (**Fig. 2**), TRP-1-specific CD4^+^ T cells were increased in IL-15^−/−^RAG^−/−^ hosts (**Fig. 5D**). These results show that, when NK1.1^+^ cells are absent due to lack of IL-15, tumor immunity is improved, and suggests that activation of Fc receptors does not contribute to enhanced tumor immunity. This also suggests that IL-15 is not absolutely required for CD4^+^ T cell homeostasis.

### Depletion of pre-mNK cells enhances tumor rejection

Next, because IL-15^−/−^ mice lack both pre-mNK cells^46^ and NK cells, and because specific depletion of NK cells has little effect on tumor rejection in our system (**Fig. 4**), we reasoned that pre-mNK cells (NK1.1^+^B220^+^CD11c^lo^GR-1^−^ cells) had a potential role in suppressing antitumor immunity. Since these cells can reduce autoimmune pathology and express MHC class II,^30^ it may be possible that naive TRP-1 CD4^+^ T cells are interacting with pre-mNK cells and receiving a signal that blocks their antitumor activity.

**Figure 6.**
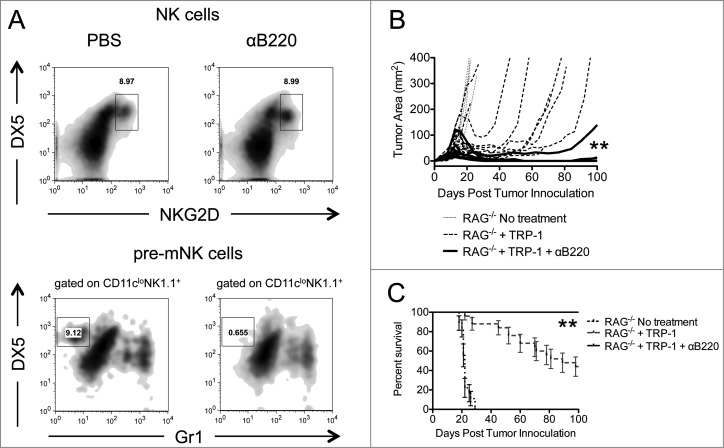
Depletion of pre-mNK cells by anti-B220 enhances the rejection of established melanoma. RAG^−/−^ mice were inoculated subcutaneously with 3×10^5^ B16.F10 on day 0 and left untreated, received ACT of 5×10^4^ TRP-1-specific CD4^+^ T cells on day 7, or received ACT on day 7 plus 200 μg of anti-B220 intraperitoneally on days 0, 7, and 14. (**A**) Splenocytes from each indicated experimental group were harvested at day 21 post tumor inoculation and analyzed for the presence of NK cells and pre-mNK cells. (**B**) Tumor area as a function of time for each replicate of each experimental group is plotted. **, *p* < 0.0001. (**C**) Percent survival for each of the aforementioned experimental groups was plotted as a function of time post tumor inoculation. Percent survival for each group is significantly different, **, *p* < 0.0001.

In an effort to determine whether pre-mNKs were responsible for the tumor growth and survival discrepancies between RAG^−/−^ animals receiving ACT alone or RAG^−/−^ animals receiving anti-NK1.1 antibodies in addition to ACT, we performed ACT in combination with anti-B220 therapy. In RAG^−/−^ lymphopenic mice, anti-B220 antibodies deplete NK1.1^+^B220^+^NKG2D^+^DX5^+^GR-1^−^ pre-mNKs but not NK1.1^+^B220^−^NKG2D^+^DX5^+^ NK cells (**Fig. 6A**). Animals receiving anti-B220 antibodies in addition to ACT experienced enhanced tumor immunity, increased survival, and fewer relapses (**Figs. 6B and C**, **Table 1**). Serum levels of INFγ were also higher in B220 depleted animals compared to other groups (*data not shown*), suggesting a stronger antitumor response. These results imply that B220^+^NK1.1^+^, presumably pre-mNK cells, may be responsible for the suppression of the rejection of established melanoma tumors by TRP-1-specific CD4^+^ T cells in our model.

## Discussion

It has long been recognized that profound lymphodepletion prior to ACT increases the efficacy of therapy.^22,23^ This effect has been attributed to the elimination of suppressor cells and cytokine sinks as well as enhanced activation and availability of APCs.^47^ Herein, we have described how depletion of NK1.1^+^ cells enhances the rejection of established melanoma by adoptively transferred TRP-1-specific CD4^+^ T cells. Others have suggested that this is due to the elimination of NK cells, which serve as cytokine sinks in the context of ACT of gp100-specific CD8^+^ T cells.^19^ However, we don't believe that this conclusion fully explains our observations, given that anti-Asialo-GM1, which depletes NK cells, does not enhance ACT of TRP-1-specific CD4^+^ T cells to the same extent as anti-NK1.1. The fact that animals receiving anti-NK1.1 therapy in addition to ACT experience vitiligo sooner than animals receiving ACT alone suggests that the adoptively transferred TRP-1-specific CD4^+^ T cells are rejecting TRP-1^+^ cells more quickly under these conditions. We also interpret the fact that NK1.1^+^ cell depletion reduces the number of relapses to mean either that TRP-1-specific CD4^+^ T cells reject melanoma cells more completely or experience exhaustion to a lesser degree in the absence of NK1.1^+^ cells^35^ as has been suggested in viral infection models.

Pre-mNK cells (IKDCs) were originally characterized by their ability to kill tumor cells.^26,27^ Taieb et al., describe IKDCs (pre-mNK cells) as the main source of IFNγ in their mouse model of melanoma. They found that treatment with imatinib mesylate (IM) and IL-2 induced the expansion of pre-mNKs in the spleen. Pre-mNKs from mice receiving IM and IL-2 therapy could kill tumor *ex vivo* and reduce tumor burden *in vivo* via a TRAIL-dependent mechanism.

Chan et al., observed that splenic pre-mNK cells could lyse the classical NK targets, YAC-1 and Ba/F3 in an NKG2D- and Ly49h-dependent manner, respectively, after they had been activated with the TLR-9 ligand, CpG oligodeoxynucleotide (ODN). Lytic activity and surface expression of NKG2D decreases between 14 and 36 h following CpG ODN stimulation. At the same time, pre-mNKs upregulate MHC II and induce CD4^+^ T cell proliferation. They never observed the same lytic activity from CpG ODN-activated, lymph node-derived pre-mNKs. They also report that IL-15 and IL-12 can induce IFNγ production by pre-mNKs.^27^

Intraperitoneal (IP) injection of the TLR-3 agonist, poly I:C, has been shown to induce the expansion of and IFNγ production by pre-mNK cells. Poly I:C-stimulated pre-mNKs were observed to delay melanoma progression in a murine model of lung metastasis.^48^

Finally, bone marrow-derived pre-mNK cells cultured in the presence of GM-CSF and stimulated with LPS could delay tumor progression after adoptive transfer into a mouse model of rhabdomyosarcoma. This tumoricidal activity was dependent upon recognition of NKG2D ligands, IFNγ production, and host effector cells.^49^

In each of the aforementioned models, pre-mNK cells were activated prior to the observation of their tumoricidal activity. To these authors' knowledge, Himoudi et al., was the only group to have previously examined the activity of unactivated pre-mNKs during tumor growth. They found that injection of freshly isolated bone marrow derived pre-mNKs delayed the growth of rhabdomyosarcoma tumors in *beige* mice.^50^ However, *beige* mice lack functional CTLs.^51^

Previous work by our group and others has demonstrated that cytotoxic CD4^+^ T cells are capable of inducing the regression of established melanoma.^10,11^ The activity of unactivated pre-mNK cells in the context of a cytotoxic CD4^+^ T cell response has not been previously investigated. Pre-mNKs are known to present antigen to CD4^+^ T cells *in vitro* and *in vivo*.^52,53^ This process requires direct contact between the pre-mNK and a tumor cell or infected fibroblast, and subsequent upregulation of MHCII as well as IFNγ and PD-L1. Thus our data suggest that TRP-1 CD4^+^ T cells could interact with MHC class II^+^ pre-mNK cells and become either exhausted or turned into regulatory T cells. Depletion of NK1.1^+^ cells then prevents this as shown by less T cell exhaustion and fewer T regulatory cells which leads to less recurrence of melanoma in our model.

Furthermore, these observations are unlikely due to Fc-receptor stimulation by the constant region of the depleting antibodies used. ACT of TRP-1-specific CD4^+^ T cells was able to reject established B16.F10 melanoma tumors and increase survival in IL-15^−/−^RAG^−/−^ hosts relative to RAG^−/−^ hosts without the addition of antibodies.

These experiments also confirm that IL-15 is not required for naive CD4^+^ T cell homeostasis or tumor rejection in our system. It has long been appreciated that IL-15 is necessary for NK and memory CD8^+^ T cell homeostasis. New evidence is emerging that IL-15 is also beneficial for memory CD4^+^ T cell homeostasis.^54-56^ However, the function of IL-15 in the setting of a naive CD4^+^ T cell response is still the subject of much debate. Some reports suggest that IL-15 supports the maintenance and development of T_reg_ cells.^56,57^ This makes sense in the context of our data. T_reg_ cells are known to dampen the antitumor response. Therefore, if IL-15^−/−^ mice could not support optimal T_reg_ cell function, we would expect our TRP-1-specific CD4^+^ T cells to reject tumors more efficiently in these hosts. Others have suggested that IL-15 protects CD4^+^ T cells from AICD and, in the presence of a TCR signal, promotes proliferation of this population.^58^ Some hypothesize that this proliferation may be due to IL-15′s capacity to interfere with the regulatory activity of T_reg_ cells.^59^ However, the CD4^+^ T cells that proliferate under such conditions have a decreased ability to produce IFNγ.^43^ While we have done no experiments to rule out increased AICD or decreased proliferation in our system, we stress that, if these phenomena are occurring, they are not sufficient to appreciably dampen the antitumor immune response. Furthermore, in the study by Chen et al., IL-15^−/−^ animals were used, and the possibility that pre-mNKs, which are absent in these mice, may suppress the expansion of naive CD4^+^ T cells was not addressed. Furthermore, the induction of T_reg_ cells by pre-mNK cells was not ruled out.

Since anti-NK1.1 and anti-B220 target multiple cell types, we cannot rule out the possibility that each antibody has depleted a different population of cells. However, we find this explanation unlikely. There are a limited number of NK1.1^+^ cell populations in RAG^−/−^ hosts: pre-mNK cells, NK cells, and, according to some investigators, ILCs. ILC-depletion by anti-Thy1.2 antibody fails to improve tumor rejection by adoptively transferred TAA-specific Thy1.1^+^CD4^+^ T cells in our model. Therefore, the cell population depleted by anti-NK1.1 antibody is very likely pre-mNK cells or NK cells. This coupled with the fact that anti-B220 (which targets pre-mNKs) enhances melanoma rejection to a greater extent than anti-Asialo-GM1 (which targets NK cells, although slightly less effectively than anti-NK1.1) following ACT suggests that pre-mNK cells are regulating the activity of the adoptively transferred CD4^+^ T cells. Finally, pre-mNK cells are IL-15 dependent.^28^ We have shown that tumor rejection is enhanced in IL-15^−/−^RAG^−/−^ mice, which have low numbers of pre-mNK cells, and that these mice also experience tumor recurrence less frequently than RAG^−/−^ mice (**Table 1**). These data strongly suggest that pre-mNK cells are responsible for suppressing antitumor immunity. However, further investigation is required to describe the precise mechanism by which this occurs.

It is reasonable to expect that the specific activity of pre-mNK cells be dictated by their activating stimuli. Chronic viral infection and cancer have similar immunosuppressive effects.^37,60^ HIV-1 infection leads to the TRAIL-mediated apoptosis of uninfected CD4^+^ T cells in NOD-SCID mice reconstituted with human PBLs.^61^ Human pDC can be transformed into IFN-producing killer pDC (IKpDC; a population with many similarities to murine pre-mNK cells) by exposure to HIV-1. HIV-1 stimulation of TLR-7 induces the upregulation of TRAIL. TRAIL expression renders IKpDC competent to lyse CD4^+^ T cells. Notably, IKpDC do not appear to express CD56, which defines a population of suppressive human NK cells, nor IFNγ, which is expressed by murine pre-mNK cells. However, this could represent a difference between human and murine homologous cell types, or a differential response by these cells to viruses and malignancies.^62^ Finally, Schuster et al., report that an NK1.1^+^NKp46^+^ population of cells kill activated CD4^+^ T cells in a TRAIL-dependent manner in a mouse model of chronic MCMV infection.^39^

Although pre-mNK cells (IKDC) were first described for their role in antitumor immunity, they have also been observed to control tolerance to self-antigens.^30^ Interestingly, melanoma licenses NK1.1^+^B220^+^CD11c^+^MHC class II^++^ pre-mNK cells to present antigen.^50,52^ Activated pre-mNK cells express the inhibitory PD-1 ligand, PD-L1, and produce the immunosuppressive cytokine, IL-10,^30^ both of which have been demonstrated to suppress antitumor immunity. Since TRP-1 is a tumor-associated antigen, it is possible that pre-mNK cells could perceive TRP-1-specific CD4^+^ T cells to be autoreactive. In this case, it is conceivable that pre-mNKs would promote tolerance to TRP-1^+^ cells, including melanoma, rather than immunity. Increasing lymphodepletion intensity prior to ACT or targeting pre-mNKs with anti-NK1.1 or anti-B220 may remove this cell, thus disinhibiting the TAA-specific CD4^+^ T cells.

Pre-mNK cells have been suggested to protect the host from autoimmunity. Activated pre-mNK cells have been shown to suppress autoimmunity in an EAE model of multiple sclerosis.^30^ Huarte et al., observed that, after administration of a tolerogenic agent, pre-mNK cells protect against EAE by killing activated CD4^+^ T cells and mature DCs. In this model, pre-mNKs upregulated PD-L1, and GzB. CD4^+^ T cell lysis was found to be perforin-dependent.^30^ Pre-mNK cells also recruited T_reg_ cells into the CNS and produced IL-10.

Furthermore, it has been shown that autoimmune prone NOD mice, which are susceptible to diabetes due to genetic mutations linked to the distal end of chromosome 7,^63^ have reduced numbers of pre-mNK cells. NOD-Lc7 mice, which have a WT distal end of chromosome 7, are not prone to diabetes and have significantly higher numbers of pre-mNK cells.^28^ Tumors are known to co-opt the immune mechanisms that protect against autoimmunity to inhibit the antitumor immune response. Therefore, successful immunotherapies often induce autoimmune pathologies, such as vitiligo.^64^

We believe that our data suggest at least one of three possible mechanisms by which pre-mNKs suppress the rejection of established melanoma by TRP-1-specific CD4^+^ T cells. First, it may be that the IFNγ produced during the normal antitumor response of pre-mNK cells upregulates PD-L1 on the tumor or other cells in a mechanism of adaptive resistance.^65^ Thus, when recently activated PD-1^+^CD4^+^ T cells come into contact with PD-L1^+^ cells, their antitumor activity will be suppressed. Second, it may be that pre-mNK cells are activated in the tumor bed, upregulate MHCII, and travel to the draining lymph nodes where they encounter the TRP-1-specific CD4^+^ T cells. There, the pre-mNKs may kill activated CD4^+^ T cells with TRAIL, or perforin and GzB. Third, they may be directly responsible for inducing chronic exhaustion of recently activated TRP-1-specific CD4^+^ T cells through PD-L1 or IL-10, as depletion of NK 1.1^+^ cells has been shown to prevent exhaustion of T cells during anti-viral immunity^35^ and tumor recurrence, as shown here.

In conclusion, our data demonstrate that pre-mNK cells may be co-opted by established tumors to suppress antitumor immunity. The specific mechanism by which this occurs, and whether a similar mechanism exists in humans has yet to be determined.

## Methods

### Mice

*Tyrp1*^*B-w*^RAG^−/−^ TRP-1-specific CD4^+^ TCR transgenic mice (B6.Cg-*Rag1*^*tm1Mom*^ *Tyrp1*^*B-w*^ Tg(Tcra,Tcrb)9Rest/J) were previously described^11^. Recombination-activating gene 1^−/−^(*Rag1*^*tm1Mom*^) mice were purchased from Jackson Laboratories. IL-15^−/−^RAG^−/−^ mice were obtained from James P. DiSanto. All mice were used in accordance with guidelines from the University of Maryland Institutional Animal Care Committee.

### Tumor lines and measurement

B16.F10 (H-2^b^) is a TRP-1^+^ spontaneous murine melanoma that was obtained from ATCC and maintained in culture media (CM) as previously described^11^. Tumors were injected subcutaneously at 3×10^5^ cells/mouse. Tumors were measured blindly with digital calipers. The perpendicular diameters were determined and multiplied to generate the area in mm^2^, as previously described^11^.

### Sorting and adoptive cell transfer

TRP-1-specific CD4^+^ T cells were sorted from spleens of donor *Tyrp1*^*B-w*^RAG-1^−/−^ TRP-1-specific TCR-transgenic male mice. Spleens were harvested and made into single cell suspensions. Erythrocytes were lysed by ACK buffer. Subsequently, cells were counted and enriched for CD4^+^ T cells by magnetic bead sorting, using a negatively selecting CD4^+^ T cell enrichment kit from Miltenyi Biotech. Enriched CD4^+^ T cells were counted, resuspended in PBS, and adoptively transferred where indicated at a dose of 5×10^4^ cells per mouse. T cells were injected intravenously through the tail vein.

### Flow cytometry

Anti-CD4 (RM4–5), anti-IFNγ (XMG1.2), and anti-TNFα (MP6-XT22) were obtained from BD Biosciences (San Jose, CA). Anti-B220 (RA3–6B2), anti-NK1.1 (PK136), anti-CD11c (N418), anti-Gr-1 (Gr-1), anti-DX5 (DX5), anti-NKG2D (CX5), anti-NKp46 (29A1.4), and anti-CD86 (GL-1) were obtained from Affymetrix/eBioscience (San Diego, CA). All flow cytometry scales are log scales, if not otherwise specified. Intracellular staining for cytokines was done with the BD Biosciences cytofix/cytoperm intracellular staining kit. All samples were run on a BD FACSCalibur (Department of Surgery, University of Maryland School of Medicine) and analyzed by FlowJo 887 Software (Tree Star, Inc.., Ashland, OR).

### Antibody therapy

Anti-NK1.1 (PK136), anti-B220 (HB220), and anti-Thy1.2 (30-H12) were purchased from BioXCell (West Lebanon, NH), where 200 μg of antibody was injected intraperitoneally on days 0, 7, and 14. Anti-Asialo-GM1 was purchased from Wako Chemicals USA, Inc.. (Richmond, VA), and reconstituted per the manufacturer's instructions, where 20 μL of antibody was injected intraperitoneally on days 2, 0, 7, and 14.

### Measurement of serum cytokines and chemokines

Serum was collected with a 1 mL syringe (B.D. Biosciences). Serum was taken from the aorta immediately after animals were euthanized and analyzed by MILLIPLEX 32-Plex assay (University of Maryland Baltimore, Cytokine Core Lab).

### Statistics

A student's unpaired *t*-test was used to compare the differences between cytokines and chemokines as indicated. Representative tumor area curves were determined by nonlinear regression and compared with an *F*-test. Survival curves were compared with a log-rank test. *p* values of 0.05 or less were considered significant. PRISM 5.0 software was used to analyze the data (GraphPad, La Jolla, CA).

## Supplementary Material

2015ONCOIMM0036R-f07-z-bw.pdf
