# Supplementary figures and images for: Depletion of B220+NK1.1+ cells enhances the rejection of established melanoma by tumor-specific CD4+ T cells
Source: Oncoimmunology. 2015 Apr 1;4(8):e1019196. doi: 10.1080/2162402X.2015.1019196 (PMC4570124; doi:10.1080/2162402X.2015.1019196)

Supplemental Data 1

A

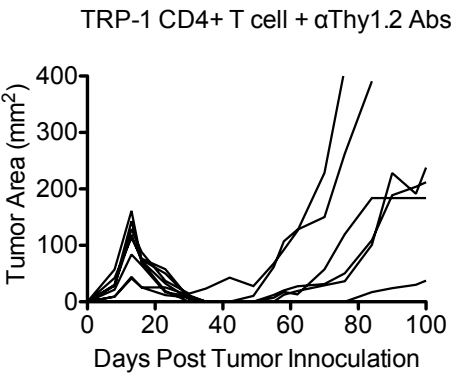

Supplement: 2015ONCOIMM0036R-f07-z-bw.pdf [file koni-04-08-1019196-s001.pdf]
